# Physical and Chemical Properties of CEM Cement Mixed with Propylene Glycol 

**Published:** 2017

**Authors:** Fereshte Sobhnamayan, Alireza Adl, Nooshin Sadat Shojaee, Zahra Zarei, Atiyeh Emkani

**Affiliations:** a *Department of Endodontics, Dental School, Shiraz University of Medical Sciences, Shiraz, Iran; *; b *Department of Endodontics, Biomaterials Research Center, Dental School, Shiraz University of Medical Sciences, Shiraz, Iran; *; c *Student Research Committee, Dental School, Shiraz University of Medical Sciences, Shiraz, Iran*

**Keywords:** Calcium-Enriched Mixture Cement, Calcium Release, Flowability, Microhardness, pH, Propylene Glycol

## Abstract

**Introduction:**

the aim of the present study was to evaluate the influence of propylene glycol (PG) on the flowability, microhardness, pH and calcium ion release of calcium-enriched mixture (CEM).

**Methods and Materials:**

CEM cement was mixed with different proportions of PG, as follows: group 1,100% CEM liquid (CL); group 2, 100% PG; group 3, 50% PG and group 4, 20% PG. For assessment of flowability, methodology of ADA Specification No. 57 was applied. For measuring microhardness, 80 cylindrical molds (6×4 mm) were filled with CEM cement and divided into 2 subgroups (4, 21 days) and tested using Vickers Test. Data were analyzed using the one-way ANOVA test and Tukey’s post hoc and student’s *t* test. In order to check pH and calcium release, the mixed cements were placed in cylindrical molds (5×2 mm). After 3, 24, 72 and 168 h, pH determined by a pH meter and the calcium release was measured by an atomic absorption spectrophotometer. Data were analyzed using the repeated measure ANOVA, one way ANOVA test and Tuckey’s post hoc test.

**Results:**

The present study showed that the presence of PG did not affect the flowability. With the elapse of time, microhardness was significantly increased in all groups except CL group. Regardless of time, samples with 50% PG showed the lowest pH value which was significantly different from other groups (*P*<0.05) and samples with 100% and 20% PG showed significantly higher calcium ion release compared to other group.

**Conclusion:**

addition of PG did not have any positive or negative effect on the flowability and pH of CEM cement but increased its microhardness in long term. Calcium ion release also increased in the concentration of 20% and 100%.

## Introduction

Calcium silicate-based cements (specifically mineral trioxide aggregate, MTA) are extremely biocompatible [1-3], capable of stimulating healing and osteogenesis and has been widely used for pulp capping, treatment of root perforations, root-end filling during apical surgery, orthograde root canal filling before apical surgery and apical barrier formations on nonvital teeth with open apices [4, 5]. The excellent biocompatibility of these materials is attributed to their alkaline pH and the potential to release calcium ions [6] .

An alkaline pH is an important factor for hard tissue induction and antimicrobial activity as the resistant endodontic bacteria such as *Enterococcus faecalis*, are destroyed at pH values above 11 [7, 8]. Despite all ideal properties of MTA, it is difficult to use this material because of its granular consistency [9]**,** slow setting time and initial looseness [10-12]. To improve manageability of MTA, researchers have mixed it with different vehicles including methylcellulose, calcium chloride, calcium lactate gluconate, KY liquid and propylene glycol (PG) [13-16].

PG is a viscous alcoholic compound with no evidence of carcinogenicity or genotoxicity that has been approved by the US Food and Drug Administration (FDA) as a safe food additive [17, 18]. Researchers mixed MTA with different proportions of PG to improve its handling and physical properties. Results showed a ratio of 20% PG significantly improved the flowability, adhesion, setting time and did not interfere with either the pH or calcium ion release [19].

In 2008, another calcium-silicate cement naming calcium-enriched mixture (CEM) cement was developed with clinical applications similar to MTA. The chemical composition of this cement is more similar to dentine than that of MTA and Portland cement [20]. This cement has antibacterial properties [21], induces the formation of dentinal bridge [22], has cytocompatibility comparable with MTA [23] and cementum-like tissues can form on its surface because of its biocompatibility [24]. Moreover this cement has a shorter setting than MTA. The major advantages of CEM cement over WMTA are better handling characteristic, lower film thickness and shorter setting time [22]. It is claimed that CEM cement sets in an aqueous environment [24] and it is able to stimulate hard tissue healing [25]. 

As PG could enhance some physical and chemical properties of MTA [26], the aim of the present study is to evaluate the effects of different proportions of propylene glycol (PG) on the pH , calcium ion release, flowability, and microhardness of CEM cement.

## Materials and Methods

In this *in vitro* study CEM cement (Biunique Dent, Tehran, Iran) was mixed with different proportion of PG (Merk, Darmstadt, Germany) and CEM liquid (CL). Samples were divided into 4 groups, as follows: Group 1, CEM+100% CL; group 2, CEM+100% PG; group 3: CEM+50% CL and 50% PG and group 4, CEM+80% CL and 20% PG.

The CL/PG ratios were determined by volume, and the powder/liquid ratio was the same for all groups (1 g powder to 0.54 mL liquid). The CEM powder was hand mixed using a spatula and a glass slab, following the condensation technique described by Nekoofar *et al.* [27].

***Cement consistency (flowability)***

The flowability of each group was determined based on the methodology described by the ADA Specification No. 57. Testing was carried out by placing 0.5±0.02 mL of cement over a smooth and flat glass slab immediately after manipulation. Three min after manipulation, another flat and smooth glass slab was positioned over the cement. A weight was then placed over the assembly. The weight placed on top of the cement (the added weight plus second glass slab) totaled 120 g. The assembly was then kept in an incubator at 37^°^C and fully saturated humidity. Ten min after the beginning of spatulation, the weight was removed and the diameter of the circle formed by the cement was measured with a digital caliper. The diameter was measured at its greatest and smallest dimensions and the mean value in millimeters was considered as a measurement of the flowability. Three tests per group were carried out. Kruskal Wallis test was used to analyze the data.

***Surface microhardness***

A total of 80 cylindrical molds made of poly methyl methacrylate with an internal diameter of 6 mm and height of 4 mm (according to ASTM E384 Standard for microhardness tests) were fabricated by CNC laser cutting (LaserProI; GCC, New Taipei City, Taiwan). CEM Cement were prepared by mixing 1 g powder with 0.54 mL associated liquid of material with different proportion of PG specimens were divided into 2 subgroups according to the time (4 and 21 days). The molds were then filled with the prepared cements by using minimal pressure [17, 28, 29] and wrapped in gauze soaked with PBS on the top and bottom of the samples and incubated at 37^°^C in 100% relative humidity for 4 and 21 days. The samples were then polished by using silicon carbide sandpaper with decreasing particle sizes of 400, 500, 800, 1000, 1200, 1500, and 2000 grit, respectively. For the purpose of facilitating indentation and minimizing the influence of sample preparation on surface microhardness, wet polishing with minimal hand pressure was used.

The surface microhardness test was performed by using a Vickers Tester (Bareiss Prufgeratebau GmbH, Oberdischingen, Germany) with a pyramidal diamond indenter by using a load of 300 g for 10 sec. According to the pilot study, this load created a clear and reliable indent in materials. Five indents were made on the polished surface of each sample at separate locations with a 2.5×d (2.5 times the mean diameter of each indent) distance between indentations and from the edge of the sample (in accordance with ASTM E384 standard for Vickers microhardness test). The Vickers microhardness value was calculated by the testing machine on the basis of the following equation in which *F* is the load in kilogram-force, *d* is the mean of the 2 diagonals in mm, and *HV* stands for Vickers microhardness value. HV=(2FSin 136/2)/D^2 ^and HV=1/854 F/D^2^.

Data were analyzed using the one-way ANOVA test for global comparison and by Tukey’s post hoc and student’s *t* test for individual comparisons.

***pH and calcium release***

The mixed cements were placed in plastic cylindrical molds measuring 5 mm in length and 2 mm in diameter in order to stimulate the dimensions of a real endodontic apical plug. The molds were weighed accurately before and after being filled with cement samples. Each tube was separately placed in plastic tubes containing 10 mL of deionized distilled water. After 3, 24, 72 and 168 h, the molds were placed in new tubes and the water in which they had been kept had its pH determined. A pH meter (827 PH lab, Metrohm, Herisau, Switzerland), previously calibrated using buffer solutions of pH 4 and 7 was used for determination of the pH of the water in which the molds had been kept. For determination of calcium ion release, CTA-3000 atomic absorption spectrophotometer (Chem Tech Analytical Instruments Limited, UK) with a calcium-specific hollow cathode lamp and Air/acetylene flame was used; calcium release was measured in mg/dL. The water in which calcium ion release was measured was the same used in the pH measurements. To prevent potential interferences from alkaline metals, standards and samples were combined with a 0.20 mol/L lanthanum and potassium chloride solution. Standard solutions containing 0.05, 0.5, 1, 1.5 and 2.0 mg L/1 of calcium were utilized to obtain calibration curve. All samples were diluting the 1:10 ratio using deionized distilled water just before absorption reading to reach to the signal of constructed calibration curve. Finally calcium release calculations were performed by means of the equation of the standard curve and this dilution factor was considered to determine the final concentrations of samples. For the *blank*, the same amount of lanthanum chloride and potassium chloride was added to the suitable volume of deionized water. For *zero* absorbance, a nitric acid solution was used. Ca^++^ ion release readings were carried out at the same periods in which the pH was determined.

Data were analyzed using the repeated measure ANOVA. As there was an interaction effect between time and materials, subgroup analysis was done. For comparison of different groups in each time, one-way ANOVA and Tukey’s post hoc tests and for comparison of each group at different time periods one sample repeated measure ANOVA/Sidak tests were used. The level of statistical significance was set at 0.05.

## Results

***Flowability***

The mean, standard deviation and significant differences in flowability (in mm) are shown in table 1. Statistical comparison showed significant differences between group 3 and 4 (*P*=0.027) and also group 2 and 4 (*P*=0.01). 

***Microhardness***

Table 2 shows the mean and standard deviation of microhardness in all groups in 4 and 21 days. In day 4 group 1 shows a significant difference with group 2, 3 and 4 (*P*=0.001).

In day 21 group 3 had the greatest bond strength which was significantly greater than group 1, 2 and 4 (*P*=0.001) but there was not a significant difference between groups 2 and 4 (*P*=0.998). Student’s *t* test showed that with the elapse of time, microhardness significantly increased in all groups (2, 3, 4) (*P*=0.001) except group 1 (*P*=0.349).

***pH***

**Figure 1:**
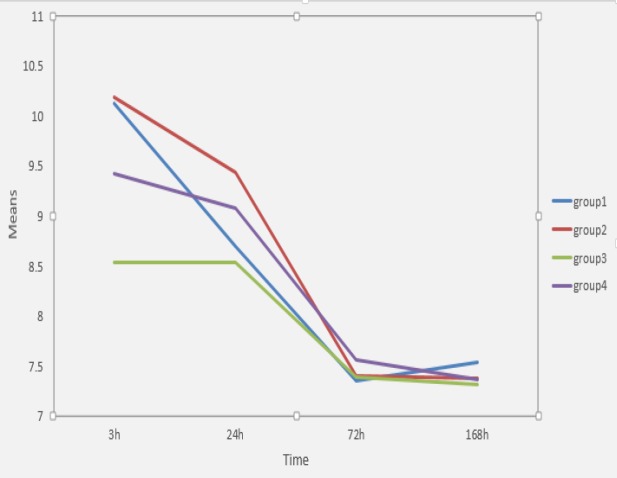
pH change in different groups over time

Table 3 presents the mean±standard deviation and statistical comparisons in the pH readings of each group at different periods. Also pH values decreased over time in all groups (Figure1). 

At 3 h the highest pH value was observed in samples with 100% PG which was significantly different from samples with 50% PG and 50% CL and samples with 20% PG and 80% CL (*P*<0.05). At 24 h, the highest pH value belonged to samples with 100% PG which was significantly different from samples with 50% PG and 50% CL and 100% CL (*P*<0.05). At 72 h, the highest pH value was observed in samples with 20 % PG and 80%CL which was significantly different from samples with 100%CL (*P*=0.042). At 168 h, no significant differences were detected between the groups studied (*P*>0.05). 

***Calcium ion release***

**Figure 2:**
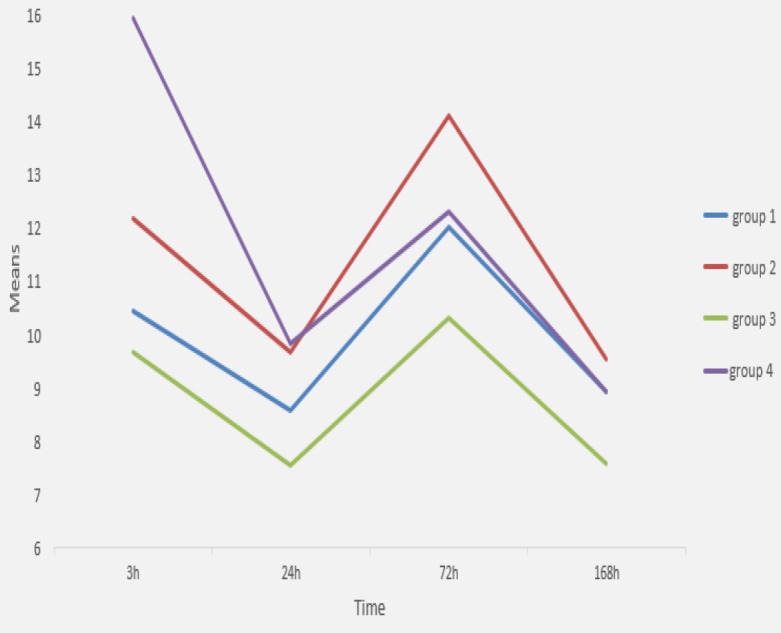
Calcium ion release in different groups over time

Table 4 shows the mean (SD) deviation and statistical comparisons for calcium ion release (in mg L/1) in all groups throughout the different periods. A fluctuant pattern was observed for all groups during time (Figure 2).

At 3 h, samples with 20 % PG and 80% CL showed the highest calcium ion release which was significantly different from other groups (*P*<0.05). At 24 h there were no significant differences (*P*>0.05) between all groups. At 72 h the highest calcium ion release belonged to samples with 100% PG and the lowest one was observed in samples with 50% PG and 50% CL group which were significantly different from each other (*P*=0.001). Analysis at 168 h showed no significant differences between the groups (*P*>0.05).

## Discussion

***Flowability***

This study revealed that mixing PG with CL as the liquid vehicle for CEM cement cannot significantly improve the flowability of this cement. Duarte *et al.* [17] showed that mixing PG with distilled water as a vehicle for MTA increased its flowability. Natu *et al.* [30] showed that PG increased the flowability of MTA when used in concentration of 20% and 50%. They claimed that increasing the flowability could contribute to achieving better adaptation to various irregularities in the root canal system and also improve the ability of the material to penetrate into perforations. On the other hand, more flowability resulted in the greater difficulties to handle the material and insert the mixture into the root canal system in clinical situations [30]. Asgary *et al.* [21] showed that CEM cement exhibited reasonable flow (14±1 mm), which were statistically different from MTA (10±0.79 mm). The present study showed that adding PG could not significantly improves the flowability of CEM cement as this material has already good flowability which is significantly better than MTA. They also claimed that the slight expansion and reasonable flow and film thickness of CEM can ensure an effective seal after setting, and reduce the subsequent leakage [21]. 

High percentage of small particles (0.5-2.5 μm) [21] resulted in better handling characteristics and possibly better flow of CEM cement. Thus it seems that there is no need to increase the flowability of this material with other vehicles such as PG although more investigation is required.

Recent studies [31-34] reported less enamel scars and more polished enamel surfaces when using aluminum-oxide discs and fiberglass burs for resin adhesive removal. Ryf *et al.* [35] found an average enamel loss of 4.1 µm after adhesive removal with tungsten carbide burs. When abrasive discs were associated to burs, enamel loss was reduced to 2.9 µm. Combination of techniques was useful on preserving dental enamel during resin adhesive removal. In the current study, DiscL caused temperature rises in the pulp chamber above 4.8^º^C in 25% of the sample (**Figure 3**). Maximum temperature rise was 7.8^º^C (Table 2), being potentially harmful to the pulp tissues. Nevertheless, DiscL allowed an optimal distinguish between enamel and resin adhesive. One could suggest intermittent use of DiscL with short time intervals, in order to avoid excessive temperature rise during resin adhesive removal.

The use of BurFGL for resin adhesive removal provoked the greatest temperature rise in the pulp chamber (8.5±1.9^º^C) (Table 2). Temperature rise with BurFGL was above 4.8^º^C in 100% of the sample (Figure 3). This outcome revealed that the use of BurFGL for resin adhesive removal is highly dangerous to the pulp tissues. Clinician must be aware of using BurFGL for resin adhesive removal in sound teeth. Time spent during resin

**Table 1:** Mean (SD) of the flow in mm for each group (Different letters are statistically significant

**Group**	**100% CL**	**100% PG**	**50% CL+50% PG**	**80% CL+20% PG**
**Mean (SD)**	46.52 (0.03)^ab^	58.05 (1.17)^a^	56.94 (1.01)^a^	44.2 (1.12)^b^

**Table 2:** Mean (SD) and statistical comparison for the microhardness of the groups studied at different periods

**Time/group**	**4 days**	**21 days**	**Time/group**
**100%CL**	31.09 (3.22)^a^	30.05 (0.91)^a^	100%CL
**100%PG**	26.93 (1.72)^b^	35.07 (1.45)^de^	100%PG
**50% CL+50% PG**	26.63 (1.43)^b^	40.04 (1.88)^b^	50% CL+50% PG
**80% CL+20% PG**	24.91 (2.01)^b^	35.19 (1.41)^ce^	80% CL+20% PG

**Table 3:** Mean (SD) and statistical comparison for the pH of the groups studied at different periods of time (Different small and capital letters indicate statistically significant difference in columns and rows respectively

**Time/group**	**3h**	**24h**	**72h**
**100%CL**	10.13 (0.89)_B_^ab^	8.7 (0.45)_C_^ab^	7.35 (0.08)_A_^a^
**100%PG**	10.19 (0.4)_A_^b^	9.44 (0.72)_A_^c^	7.4 (0.09)_B_^ab^
**50% CL+50% PG**	8.54 (0.63)_A_^c^	8.54 (0.14)_A_^a^	7.39 (0.22)_B_^ab^
**80% CL+20% PG**	9.42 (0.51)_A_^a^	9.08 (0.16)_A_^bc^	7.56 (0.2)_B_^b^

**Table 4:** Mean (SD) and statistical comparison for calcium release (mg L/1) in the groups, at different periods of time (Different small and capital letters indicate statistically significant difference in columns and rows respectively

**Time/group**	**3h**	**24h**	**72h**	**168h**
100%CL*	10.44 (3.1)_AB_^a^	8.60 (4.14)_AB_^a^	12.03 (2.08)_A_^a^	8.94 (0.97)_B_^a^
100%PG**	12.18 (2.47)_AB_^a^	9.67 (1.63)_BC_^a^	14.1 (2.47)_A_^b^	9.55 (2.22)_C_^a^
**50% CL+50% PG**	9.67 (2.54)_AB_^a^	7.56 (1.45)_A_^a^	10.33 (0.71)_B_^a^	7.58 (1.09)_A_^a^
**80% CL+20% PG**	15.94 (3.99)_A_^b^	9.85 (2.3)_B_^a^	12.3 (2.2)_AB_^a^	8.94 (2.96)_B_^a^

adhesive removal had a high correlation with temperature rise in the pulp chamber (*r*=0.826). Procedure time determined 68% of the temperature rise. BurFGL demanded up to 44 sec to be completed and showed 11.8^º^C of maximum temperature rise. The greater was the time spent during adhesive removal, the greater was the temperature rise in the pulp chamber. Temperature rises and time spent followed the same sequence of increase among the adhesive removal techniques (Table 2). 

This *in vitro* study has clear limitations. In *in vivo *conditions, the blood circulation into the pulp chamber and the fluid movement into the dentinal tubules interfere in the heat conduction inside the tooth and can produce a different temperature response to the resin adhesive removal process [35]. In addition, the surrounding periodontal tissues promote the dispersion of heat, limiting the increase in the pulp temperature. The temperature rise might be higher in young teeth due to the greater volume of the pulp and to the thinner thickness of the dentin. In older teeth, the deposition of secondary dentin is enhanced. 

Outcomes of the present study enrich the current knowledge on temperature rises in the pulp chamber caused by dental procedures for resin adhesive removal. The use of BurH-cool, BurH and BurL spent a shorter time and produced low temperature rises. Differently, DiscL and BurFGL lasted longer during adhesive removal and caused temperature rises above 5.5^º^C, especially the latter. In addition to clinician preference, choice of resin adhesive removal techniques must rely on effective enamel cleaning with low temperature rise. Alternative procedures could be thought towards adhesive removal, such as ultrasonic with abundant water-cooling. However, if used in dry mode, at least for removal of metallic posts, injurious heat.

***Microhardness***

Microhardness tests are used for evaluating the quality and progression of the hydration process and as an indicator of the completeness of the setting process. It is based on evaluating the resistance of materials to deformation [36, 37]. In the present study mixing CEM cement with 100% PG resulted in a creamy and loose mixture that was not set in the first three hours. Adding PG to the CL significantly decreased the microhardness of CEM cement in the first 4 days but in the 21 day it has a positive effect on the microhardness of CEM cement. This is partly in agreement with the result of Natu *et al.* [30] although it is not directly comparable. He showed that Mixing MTA with water and PG at different concentrations resulted in a smooth and creamy mixture. The addition of PG reduces the amount of water available for the hydration reaction, resulting in longer initial setting times and thus decrease the microhardness. Duarte *et al.* [17] also showed that adding PG to MTA increased its setting time and increasing the PG proportion interfered with the setting time of MTA. It seems that increasing the setting time of MTA in the initial hour or even days could decrease the microhardness of the material. Up to our knowledge there is no study on the microhardness of MTA and CEM cement when mixed with PG in order to compare with the present study. More studies are needed to evaluate the effect of PG on the properties of this cement in longer time periods.

***pH and calcium ion release***

It has been suggested that the high pH and released calcium and phosphorus ions are required for a material to stimulate mineralization in the process of hard tissue healing [38]. The excellent biocompatibility of MTA, hydroxyapatite and other calcium-containing materials may contribute to their ability to release calcium ions which react with phosphate ions of body tissue fluid, resulting in hard tissue formation. Sarkar *et al.* [6] reported that MTA in synthetic tissue fluid produced precipitates with similar composition and structure to hydroxyapatite. Similarly Asgary *et al.* [39] showed that CEM had the ability of hydroxyapatite formation in synthetic tissue fluid and normal saline solution. Furthermore, increasing pH levels contributed to antibacterial activity; a critical factor in the formation of a mineralized tissue barrier [40]. 

The methodology used in the present study was similar to those used by Duarte *et al.* [41] and Santos *et al.* [42]. An atomic absorption spectrophotometer with a calcium-specific cathode lamp, a highly reliable device was used to measure calcium release [43]. Plastic tubes instead of extracted teeth were used for the study as it is difficult to standardize the foramen opening. Once the reading was performed, the tubes were again immersed in another flask containing distilled water, avoiding therefore the ionic saturation that would interfere in the final outcomes [43] .

In the present study, for all groups, the maximum amount of pH value was observed at 3 h which decreased until it was stabilized after 72 h. However, Abbaszadegan *et al.* [44] and Ghazvini *et al.* [45] showed that the maximum increase of pH occurred up to 24 h and then remained the same or slightly increased over time. This discrepancy may be attributed to the different experimental set up that has been used in these studies. In the present study molds with 2 mm internal diameter and 5 mm height were chosen to stimulate the apical plug situation but Abbaszaedagn *et al.* [44] used rings with 12 mm internal diameter and 2 mm height for evaluating solubility and pH value of CEM cement in different time intervals. Ghazvini *et al.* [45] used plastic tubes 10 mm long and 1.5 mm in diameter and also used different storage methodology to evaluate the pH value. 

To the best of our knowledge no other study has evaluated the effect of PG on the chemical properties of CEM cement to compare with the present study. However, Duarte *et al.* [17] conduct a similar study on the effect of PG on the pH value of MTA. They concluded that the addition of PG to MTA increased the pH value only at the initial periods (3 h).

Natu *et al.* [30] also reported that when different proportions of PG were added to distilled water (20% and 50%), no significant increase was observed in the pH value of MTA samples at any periods (3, 24, 72 and 168 h).

According to the results of the present study, at 3 h, only 20% PG had a positive effect on calcium ion release from CEM cement. However, at 24, 72, and 168 h adding PG had no positive effect on the calcium ion release. Moreover, a fluctuant pattern of calcium ion release was observed in all groups; with higher values in 3 h and 72 h and lower values in 24 h and 168 h. This finding is partly in accordance with Ghazvini *et al.* [45] who showed that the rate of calcium release in CEM samples was constant from 1 h to 24 h then decreased at 48 h and again increased to its maximum rate in 28 days.

Previous studies on the effect of PG on the calcium ion release of MTA have shown different results. In the study by Natu *et al.* [30], calcium ion release increased over time and after 168 h, the highest amount of Ca^2+^ was observed in 20% PG. On the other hand Duarte *et al.* [17] showed that calcium ion release decreased over time and different proportions of PG had positive effect only at 3 and 168 h

## Conclusion

Under the limitation of this study, some improvement in the microhardness and calcium ion release of CEM cement was detected when mixed with PG. Meanwhile no negative effect was observed in the pH value and flowability. Therefore more investigations are recommended to evaluate the effect of PG on the other physical and chemical properties of this cement.
